# Usefulness of Transurethral Incision as a Primary Treatment of Ureterocele in Children

**DOI:** 10.1155/DTE.2.189

**Published:** 1996

**Authors:** Ichiro Takeuchi, Katsuya Nonomura, Koichi Kanagawa, Tetsufumi Yamashita, Hidehiro Kakizaki, Toshiaki Gotoh, Tomohiko Koyanagi

**Affiliations:** Department of Urology Hokkaido University School of Medicine North-15, West-7 kita-ku Sapporo 060 Japan

## Abstract

Seventeen patients (11 girls and 6 boys, with bilateral cases in 4 for a total 21 units), in whom ureterocele was diagnosed at from 5 days to 11 years old, were treated with transurethral incision as a primary treatment. Urinary tract infection was the most common presenting sign in 8 patients. A voiding disturbance was observed in 10 patients. Seven units were diagnosed as intravesical ureteroceles of a single system and 14 units as ectopic ones (12 associated with the duplex system and 2 with a single system). De novo reflux occurred in 12 units, but in 5 units resolved spontaneously. All 5 refluxes in mate units improved, and 2 refluxes in the contralateral ureter also disappeared. The control of infection became easy in all patients except for one with a sphincteric ureterocele. Split renal function on ^99m^Tc-diethylenetriamine pentaacetic acid scintigraphy was prominently improved in 5 systems (35.7%) and normal kidney growth was obtained in 11 systems (78.6%). A total of 13 affected units (68.4%), including 7 units (6 intravesical and 1 ectopic) for which transurethral incision seemed to have been the sole necessary treatment, were saved. We believe that transurethral incision of ureteroceles is a very useful technique as a primary treatment for all types of ureteroceles in children of all ages.


*Diagnostic and Therapeutic Endoscopy*, 1996, Vol. 2, pp. 189–196
Reprints available directly from the publisher
Photocopying permitted by license only

© 1996 OPA (Overseas Publishers Association)
Amsterdam B. V. Published in The Netherlands
by Harwood Academic Publishers GmbH
Printed in Singapore

### Usefulness of Transurethral Incision as a Primary Treatment of Ureterocele in Children

ICHIRO TAKEUCHI, KATSUYA NONOMURA, KOICHI KANAGAWA, TETSUFUMI YAMASHITA, HIDEHIRO KAKIZAKI, TOSHIAKI GOTOH, and TOMOHIKO KOYANAGI

*Department of Urology, Hokkaido University School of Medicine, Sapporo, Japan*

*(Received June 27, 1995; in final form August 24, 1995)*

Seventeen patients (11 girls and 6 boys, with bilateral cases in 4 for a total 21 units), in whom ureterocele was diagnosed at from 5 days to 11 years old, were treated with transurethral incision as a primary treatment. Urinary tract infection was the most common presenting sign in 8 patients. A voiding disturbance was observed in 10 patients. Seven units were diagnosed as intravesical ureteroceles of a single system and 14 units as ectopic ones (12 associated with the duplex system and 2 with a single system). De novo reflux occurred in 12 units, but in 5 units resolved spontaneously. All 5 refluxes in mate units improved, and 2 refluxes in the contralateral ureter also disappeared. The control of infection became easy in all patients except for one with a sphincteric ureterocele. Split renal function on $^{99m}$Tc-diethylenetriamine pentaacetic acid scintigraphy was prominently improved in 5 systems (35.7%) and normal kidney growth was obtained in 11 systems (78.6%). A total of 13 affected units (68.4%), including 7 units (6 intravesical and 1 ectopic) for which transurethral incision seemed to have been the sole necessary treatment, were saved. We believe that transurethral incision of ureteroceles is a very useful technique as a primary treatment for all types of ureteroceles in children of all ages.

**KEY WORDS:** Renal function, transurethral incision, ureterocele, vesicoureteral reflux

### INTRODUCTION

The ureterocele is a cystic dilation of the terminal portion of the ureter and causes various clinical symptoms. Several treatments for ureteroceles have been reported. Many urologists have reported successful endoscopic incision of ureteroceles without de novo reflux as a primary treatment (1–3), whereas total open operations are still widely supported for the ectopic ureterocele (4–6). We previously advocated the usefulness of transurethral incision of ureteroceles, including ectopic ones (7–9). Recently, reports recommending endoscopic incision as a primary treatment have increased, especially in infants (10–12). Endoscopic incision of ureterocele is a less invasive technique to improve the drainage of the upper urinary tract and may facilitate elective surgery, but involves the risk of reflux. This report presents our experience with transurethral incisions of ureteroceles as a primari treatment in 17 patients.

Address for correspondence: Ichiro Takeuchi, Department of Urology, Hokkaido University School of Medicine, North-15, West-7, kita-ku, Sapporo, 060, Japan.

### PATIENTS AND METHODS

Between 1980 and 1993, ureteroceles were diagnosed in 22 children in our clinic, and 17 (21 units) of these patients were treated with transurethral incision (TUI) as a primary treatment. The ages at diagnosis of the 17 patients (11 girls and 6 boys) ranged from 5 days to 11 years (median 5 months). The majority of these children (70.6%) were younger than 1 year old at diagnosis (Fig. 1). Of the 6 boys, 4 were younger than 3 months old at diagnosis. In this series, intravesical ureteroceles were diagnosed in 5 patients (2 cases bilateral for a total of 7 units), and ectopic ureteroceles were diagnosed in 12 patients (2 cases bilateral for a total of 14 units). All intravesical ureterocele units were associated with a single system and 12 of 14 ectopic ureterocele units with the duplex system. These classifications were according to the definition of the American Academy of Pediatrics, Section on Urology (13). All patients underwent a voiding cystourethrography (VCU) and intravenous pyelography (IVP) for evaluation of the urinary tract and the voiding status before endoscopic procedures. $^{99m}$Tc-

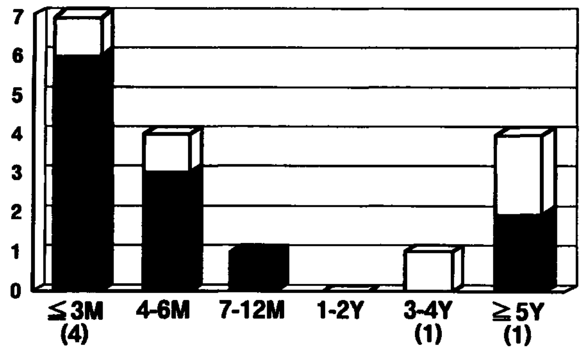

**Figure 1** Patient age at diagnosis: solid column: ectopic ureteroceles; open column: intravesical ureteroceles. Parentheses indicate a number of boys.

diethylenetriamine pentaacetic acid (DTPA) renal scintigraphy was also performed in the majority of patients. Glomerular filtration rate (GFR, milliliters per minute) and split renal function (SRF, percentage) were calculated based on Gate's method (14,15). Ultrasonography, computed tomography, and magnetic resonance image were performed in some patients for further anatomical evaluation. After the endoscopic incision, all patients were re-evaluated every 3 to 6 months, by VCU, IVP, and $^{99m}$Tc-DTPA scintigraphy.

### Technique of Endoscopic Surgery (Fig. 2)

An Olympus infantile resectscope, 10 Fr. in size, was used for TUI of ureteroceles. In intravesical ureteroceles, the incision was started from the caudal base of the cele and transversely extended a few millimeters in width by using a knife electrode. In ectopic ureteroceles, a longititudal incision was added to the urethrally extended caudal end of the cele. It is necessary to be careful that the remnant of the distal lip does not work as a valve-like structure and that excess deep cuts do not injure the bladder neck or the urethral wall.

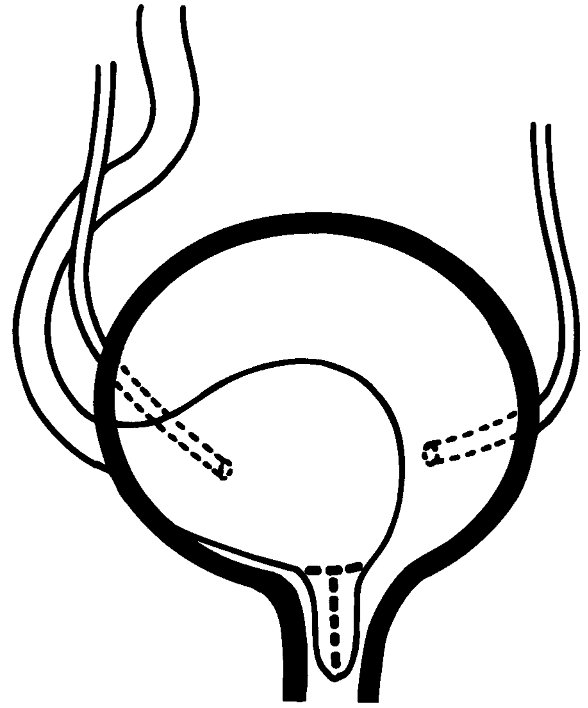

**Figure 2** Technique of TUI: T letter of broken lines represents a incisional line of cele.

### RESULTS

### Clinical Manifestations (Table 1)

Urinary tract infection (UTI) was the most common presenting sign in 8 patients, including 1 patient with prolapse of the ureterocele. Other symptoms were abdominal mass in 4, including 2 with UTI, and hematuria in 1. Prenatal hydronephrosis was seen in 4 instances, and we confirmed it postnatally. Voiding disturbances were observed in 10 patients (1 intravesical and 9 ectopic including 4 cecoureteroceles), in which the outlet obstruction was caused by the valvular effect of the caudal lip and luminal dilation of the cele during voiding. Although 5 patients (29.4%) had UTIs after incision, the control of infection was easy in all patients except one with a sphincteric ureterocele. A voiding disturbance was not observed after incision in any patients.

### Vesicoureteral Reflux (Table 2)

Three cases of vesicoureteral reflux (VUR) (14.3%) were noted in affected units, 5 (41.7%) in mate units and 2 (15.4%) in contralateral units. All these refluxes were observed in patients with ectopic ureteroceles. Eversion of the ureterocele, which was confirmed by VCU and/or endoscopy, was noted in 10 units (3 intravesical and 7 ectopic). After TUI of the ureterocele, de novo ureteral

**Table 1** Clinical Manifestations

| | *No. of Patients* |
|---|---|
| UTI | 8 ( 5 ) |
| Abdominal mass | 4 ( 4* ) |
| Prenatal hydronephrosis | 4 ( 3 ) |
| Hematuria | 1 ( 0 ) |

No. of ectopic ureteroceles is shown in parentheses.
* Including two with UTI.

**Table 2** Presence of VUR

| | *Pre-TUI* | *Post-TUI* |
|---|---|---|
| Ectopic ureterocele | | |
| Affected units | 3/14 | 13/14 (4) |
| Mate units | 5/12 | 5/12 |
| Contralateral units | 2/10 | 0/10 |
| Intravesical ureterocele | | |
| Affected units | 0/7 | 2/7 (1) |
| Contralateral units | 0/3 | 0/3 |

No. of patients whose refluxes spontaneously resolved is shown in parentheses.

reflux occurred in 12 units (2 of 7 intravesical, 28.6%; 10 of 11 ectopic, 90.9%; total 12 of 18, 66.7%), excluding 3 refluxes that had persisted before incision, but 5 (1 of 2 intravesical, 50.0%; 4 of 10 ectopic, 40.0%: total 5 of 12, 41.7%) resolved spontaneously. All 5 refluxes in the mate ureter improved and 2 refluxes in contralateral units disappeared in both units.

### Individual Kidney Function

On IVP, hydroureteronephrosis was improved in 9 systems (3 of 7 intravesical, 42.9%; 6 of 14 ectopic, 42.9%; total 9 of 21, 42.9%). In particular, the upper units in the duplex system were visualized in 2 systems. $^{99m}$Tc-DTPA renal scintigraphy of 14 affected kidneys revealed that improvements or normal kidney growths were obtained in 11 (2 of 3 intravesical, 66.7%; 9 of 11 ectopic, 81.8%; total 11 of 14, 78.6%) (Fig. 3A). The improvement of GFR in patients younger than 1 year old was especially notable (Fig. 3B). In addition, SRF was prominently improved in 5 systems. Two nonfunctioning kidneys in a single system (1 intravesical and 1 ectopic) were nephrectomized after incision of the cele and were histopathologically diagnosed as multicystic and dysplastic.

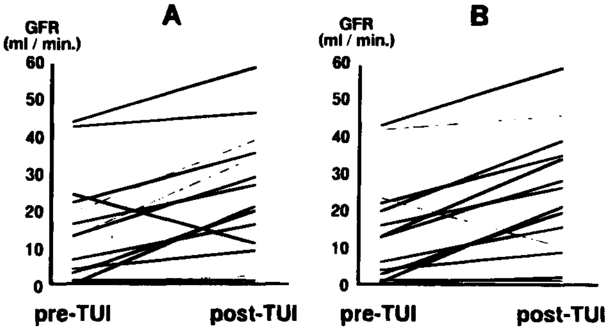

**Figure 3** Effects of TUI on kidney function: GFR of affected system. A. Type distribution at TUI: thick line, ectopic ureterocele, broken line; intravesical ureterocele. B. Age distribution at TUI: thick line, ≤1 year; broken line; >1 year.

### Treatment following TUI

Open definitive reconstructions were performed in 12 units (1 intravesical and 11 ectopic). Heminephroureterectomy was performed in 4 patients with no functioning affected kidney, and twin ureteroneocystostomy (all units were ectopic) was done in the other 6 patients with a functioning kidney (Table 3). Surgical intervention was not required in the contralateral systems except in 2 patients with bilateral ectopic ureteroceles, since all contralateral refluxes had disappeared at the definitive operation. Of the remaining 9 units, 7 (6 intravesical and 1 ectopic) were judged to need no further surgical treatment. TUI and the subsequent elective renal-conserving operations were successful in preserving the function of 13 affected units at least (6 of 7 intravesical ureterocele, 85.7%; 7 of 12 ectopic ureterocele, 58.3%; total 13 of 19, 68.4%). One patient with bilateral ectopic ureteroceles and persistent reflux after TUI underwent twin ureteroneostomy. This is the only patient whose unilateral renal function deteriorated postoperatively. Another 2 units can be followed without any deleterious effect from TUI. If these patients have febrile episodes caused by UTI in the future, elective surgery will be performed at that time. Renal functions will be preserved in both units.

### Representative Case Presentation

### *Case 1 (Fig. 4)*

A 1-month-old boy was referred for prenatal hydronephrosis discovered by ultrasonography in utero. Urinalysis was normal. IVP revealed bilateral poorly visualizing kidneys with moderate ectatic ureters and a round "cobra-head" deformity in the bladder base (Fig. 5A). VCU also showed bilateral ureteroceles as filling defects at the base of the bladder. Eversion of the right ureterocele was found in the voiding phase but was not associated with VUR. These radiological findings suggested bilateral intravesical ureteroceles in a single urinary system. Cystourethroscopy at 1 month of age confirmed the presence of bilateral intravesical ureteroceles. When the bladder was filled, the ureteroceles were

**Table 3** Definitive operative procedure

| | *No. of Units* |
|---|---|
| Ectopic ureterocele | |
| Twin ureteroneocystostomy | 6 |
| Heminephroureterectomy | 4 |
| Nephroureterectomy | 1 |
| Intravesical ureterocele | |
| Nephroureterectomy | 1 |
| Total | 12 |

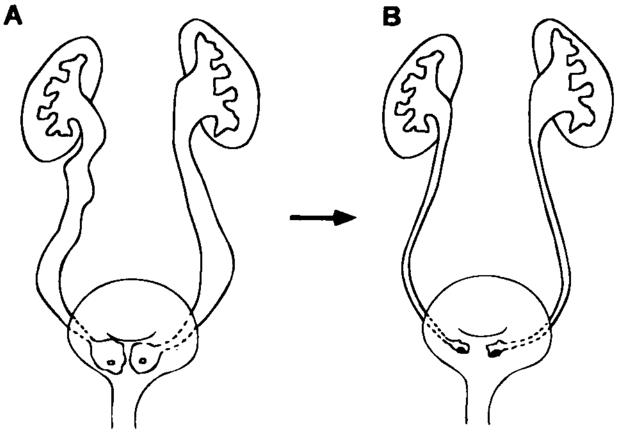

**Figure 4** Schematic illustration of case 1. A. Pre-TUI. B. Post-TUI.

collapsed and then everted. Nonetheless, endoscopic incision was performed. Bilateral hydronephrotic collecting systems dramatically improved, and the filling defects had disappeared on a postoperative IVP after TUI (Fig. 5B). Although de novo unilateral VUR temporarily appeared, this reflux had resolved spontaneously by 6-month follow-up. GFR estimation on renal scintigraphy was promptly improved by this TUI (preincision 33ml/min; postincision 75 ml/min) (Fig. 6). He has been free from renal deterioration and UTI without medication.

***Comment***

Bilateral normal kidney growths were obtained as a result of the improvement of urinary drainage. This child has not experienced UTI. The appearance of the de novo reflux was temporary. These data suggest that TUI is very useful for children with ureteroceles, especially for those younger than 1 year old in whom improvement of renal function could be expected. TUI can be the definitive treatment in this patient.

***Case 2 (Fig. 7)***

An 11-year-old girl presented to us with recurrent episodes of febrile UTI. IVP showed a representative drooping-lily sign of the left kidney, which strongly suggested the duplex system. No marked abnormality was found in the bladder (Fig. 8). VCU demonstrated VUR to the left upper and lower systems as well as a saccular structure in the urethra which obstructed the urinary flow as it was filled with urine (Fig. 9). These radiographic findings suggested the presence of cecoureterocele associated with the duplex system. On endoscopy, the ureteral orifice of the lower unit was found at the orthotopic position and the ectopic opening of the upper unit was located just proximal to the bladder neck. The cecal lumen protruded from the bladder neck and extended to the whole length of the urethra. Cecal extension of ureterocele was longititudally incised until no hold of the knife electrode was felt at its caudal extension. Postoperatively, marked

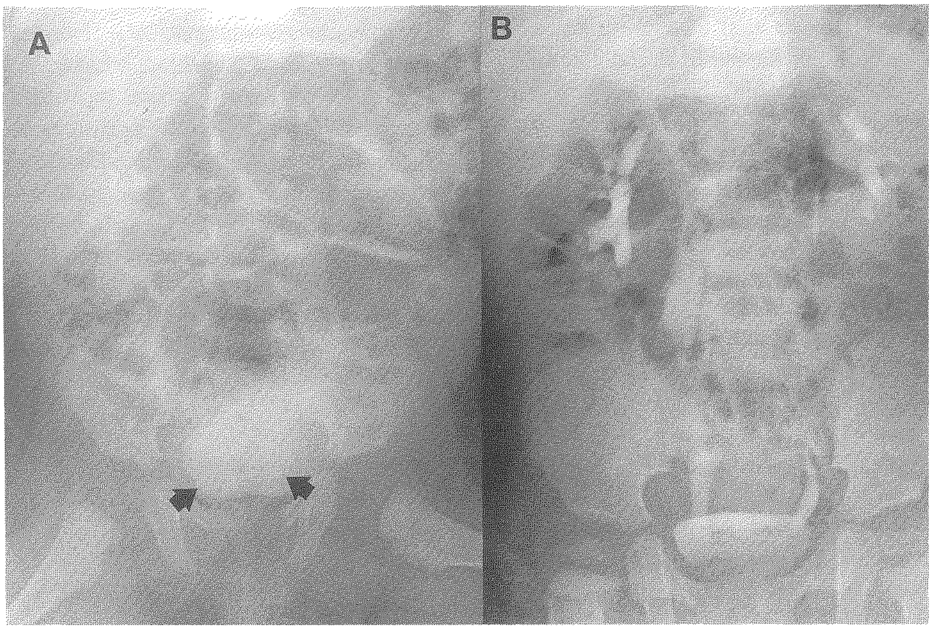

**Figure 5** IVP findings in case 1. A. Preoperative IVP: poorly visualized kidneys with ectatic ureters and round filling defects in the bladder base were revealed (arrow) B. Postoperative IVP: bilateral hydronephrosis dramatically improved and filling defects disappeared after incision.

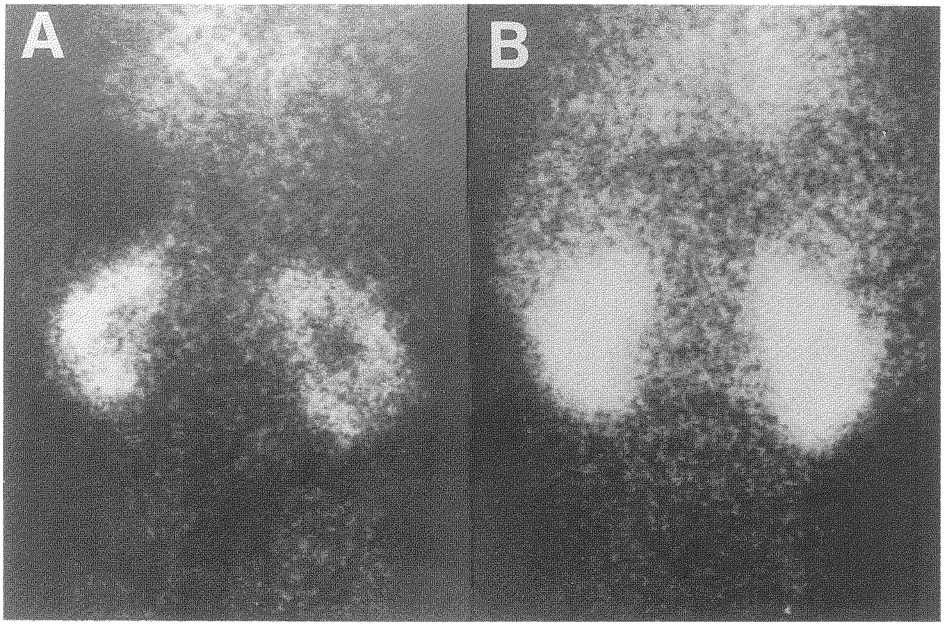

**Figure 6** $^{99m}$Tc-DTPA renal scintigram in case 1. A. Pre-TUI. B. Post-TUI. Renal uptake was prominently improved after TUI.

improvement in urinary flow as well as downgrading in ureteral reflux to a lower moiety were noted on VCU (Fig. 10). Since then, she has not experienced a single episode of UTI.

***Comment***

This patient had a belatedly diagnosed case of cecoureterocele at an older age. Although functional improvement of the left upper kidney might not be expected in this patient, UTI is under control despite persistent reflux after TUI, a product of relieving urethral obstruction from cecoureterocele. If elective surgery is ever needed, no further manipulation of the urethra will be needed.

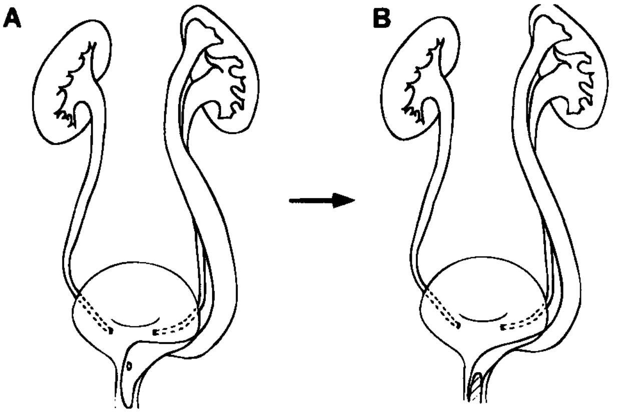

**Figure 7** Schematic illustration of case 2. A. Pre-TUI. B. Post-TUI.

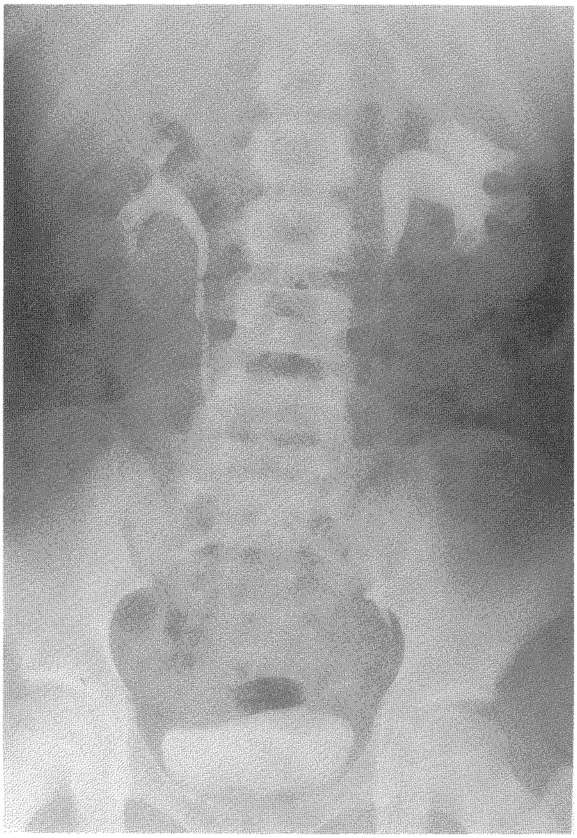

**Figure 8** Preoperative IVP findings in case 2. IVP showed drooping-lily sign of the left kidney, which strongly suggested a duplex system. No marked abnormality was found in the bladder.

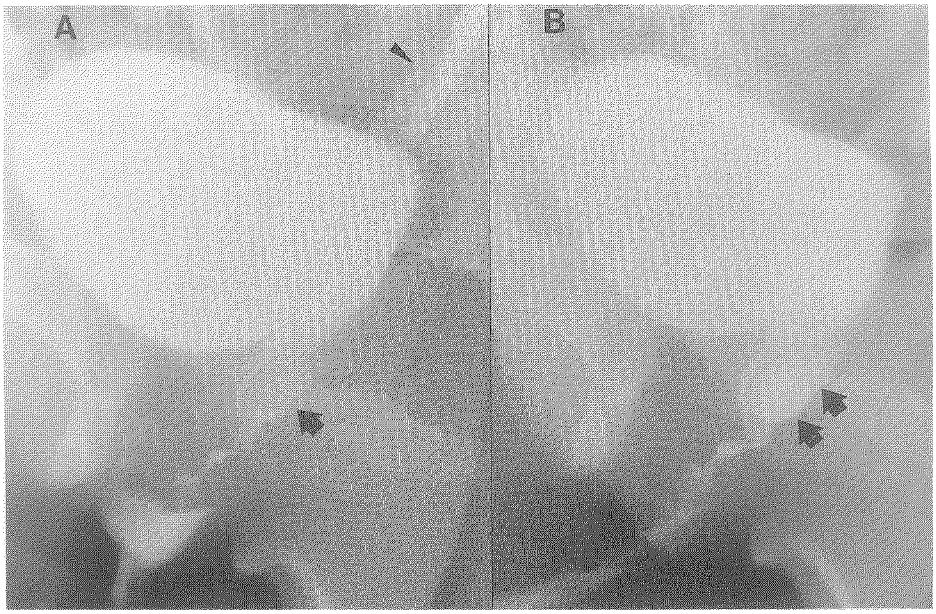

**Figure 9** Preoperative VCU findings in case 2. VCU demonstrated vesicoureteral refluxes to both units. (A: arrowhead) There is a saccular filling defect (arrow) in the urethra (A), which obviously obstructed the urinary flow as it is filled with urine. (B: two arrows)

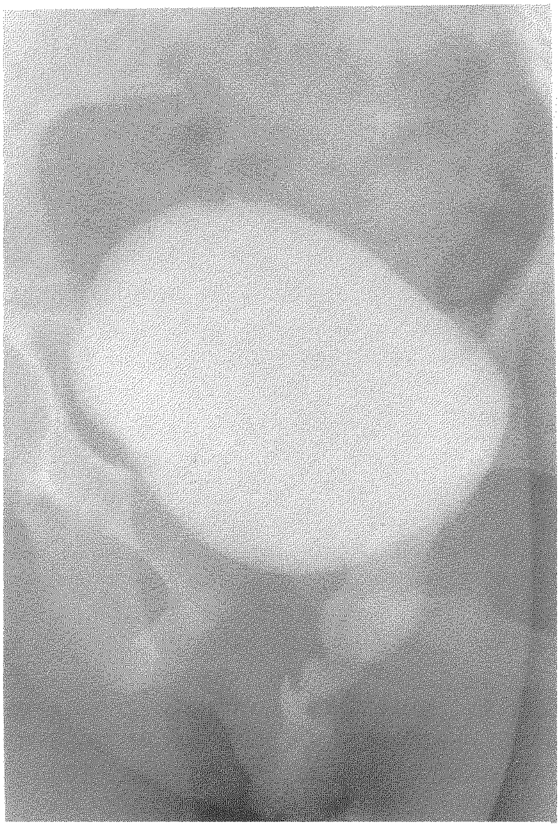

**Figure 10** Postoperative VCU findings. VCU demonstrated the downgrading of VURs in both units and voiding difficulty was clearly improved.

### DISCUSSION

The advent of fine miniature resectscopes has made endoscopic treatment for infants easier. We performed endoscopic examination in the younger children as this examination was essential for diagnosis of their urological conditions (9,16). In addition, recent diagnostic developments, including prenatal ultrasonography, have increased the early detection of various urological anomalies. Actually 6 of 7 cases of ureterocele during the last 5 years were diagnosed in patients younger than 1 year old, and 3 of these were found without infection by prenatal ultrasonography. This trend makes it essential that we choose the appropriate procedure for treatment of ureteroceles, including ectopic ones, because open surgical treatment is not easy in children, especially infants, either technically or anesthesiologically. Endoscopy for the definitive diagnosis and the incision as the primary treatment of the ureterocele can be performed simultaneously with minimal risk of hemorrhage in less than 1 hour. In this series, 6 patients in whom ectopic ureteroceles were diagnosed were younger than 3 months old and half of them were boys, a feature different from previous papers reporting higher incidence in girls (11,17). Our experience of prenatally detected VUR also shows a predominance of boys over girls (18). The advent of prenatal ultrasonography in detecting uropathies would certainly alter sexual differences of these anomalies in coming years.

Endoscopic incision of ureteroceles is said to inevitably involve the risk of VUR. In this series, a high percentage of de novo refluxes (66.7%), especially in ectopic ureteroceles (90.9%), occurred, but otherwise spontaneous resolution was obtained in 5 of 12 units (41.7%). In 9 of 10 refluxing units, including 3 in which refluxes had persisted before endoscopic incision, elective surgery was performed.

However, during the interval between TUI and definitive surgery, control of infection became easy. Additionally, we performed percutaneous nephrostomy at the same time in 5 units to examine the obstruction after incision, and a pressure flow study was carried out a few days after incision. In all units, except one sphincteric ureterocele, the nephrostomy could be removed soon because no obstruction was demonstrated urodynamically in the affected units after incision. These data suggested that endoscopic incision improved the drainage of the affected systems and that an ureteral obstruction of the upper tract was caused by the ureterocele itself. Moreover, TUI improved the voiding difficulty in children with ectopic ureteroceles. For these reasons, we believe that the benefits obtained through improvement of urinary drainage by endoscopic incision and against infection outweigh the risks of de novo reflux.

Another important clinical point is the functional recovery of the affected kidney during the follow-up period. $^{99m}$Tc-DTPA renal scintigraphy has special merits for its calculation of GFR and SRF even in patients whose affected kidney function has severely deteriorated. Recently, we have used disodium mercaptoacetoglycol-glycol-glycin ato-oxotechnetate ($^{99m}$Tc-MAG3) instead of $^{99m}$Tc-DTPA as a more potent renal uptake material. Remarkable improvement of SRF in 5 systems and normal kidney growths of GFR in 11 systems (78.6%) were obtained by the evaluation of the renal function on $^{99m}$Tc-DTPA scintigraphy. As a result, the time of the definitive surgery could be delayed without deterioration of renal function. These results also encouraged us to conclude that the benefits from decreased urinary obstruction and improvement in renal function outweigh the risks of de novo reflux.

Currently many urologists try to preserve the affected units whenever possible since the age at diagnosis is becoming younger, and diagnosis occurs in many patients before the aggravating effects of urinary infection (11,12). In this series, 13 affected units (6 of 7 intravesical ureterocele, 85.7%; 7 of 12 ectopic ureterocele, 58.3%; total 13 of 19, 68.4%) could be preserved including 7 units (6 intravesical and 1 ectopic), which might need no further treatment after endoscopic incision. Another 2 patients were followed with no further episode of breakthrough infection, and it might be possible to preserve both affected units as long as they do not cause clinically detrimental problems. Endoscopic incision as an initial treatment for ureteroceles appeared to be very useful for the preservation of the affected unit.

In this series, all 5 refluxes in mate ureters decreased and 2 refluxes in contralateral units disappeared following the improvement in voiding difficulty caused by the ureterocele itself after incision. These results suggest that endoscopic incision serves for the appropriate urinary condition at the forthcoming definitive surgery. Moreover, no surgical manipulation of the distal portion from the bladder neck to the urethra was necessary at elective surgery in ectopic ureteroceles since the treatment of the cecal lip deformity of the ureterocele was already completed. Reduction of the dilated ureter, which technically facilitates the reconstruction of the affected system including adjoining units, might be expected. Although management of VUR and the ureterocele itself remains controversial at the definitive surgery,[19,20] endoscopic incision can simplify these complex problems. We also believe that definitive surgery should be considered only when reflux persists and compromises renal function by causing uncontrollable UTI. This series includes the sole patient with decreased GFR after TUI (Fig. 3B). Despite his stable condition after TUI for bilateral ectopic ureteroceles, bilateral twin ureteroneostomies were undertaken for the presence of ureteral reflux and failed. Retrospectively, his condition could have been observed expectantly as his voiding was smooth and urinary infection was well under control after TUI.

Age at treatment is also a very important factor in deciding on the management of the ureterocele. Although many urological surgeons advocate TUI as a primary treatment in infants or when the ureterocele is intravesical, few reviews support its usefulness for ectopic ureteroceles in older children. Blyth et al.[12] mentioned that endoscopic incision should be attempted when the upper pole kidney had a functioning parenchyma or affected units were drained by a single ureter in older children. Although improvement of the affected unit's function might not be expected in older children, endoscopic incision serves for the appropriate urinary condition at the definitive surgery and prevents future urinary infection. TUI should be carried out first even in older children.

It is not easy to determine which treatments should be performed for individual patients with ureteroceles, since they have various clinical features caused by the ureterocele itself. TUI appears to make this choice easier. We believe that TUI of ureteroceles is a very useful technique as a primary treatment for all types of ureteroceles in children of all ages.

### REFERENCES

1. Monfort G, Morrisson-Lacombe G, Conquet M. Endoscopic treatment of ureteroceles revisited. J Urol 1985;133:1031–1033.
2. Tank ES. Experience with endoscopic incision and open unroofing of ureteroceles. J Urol 1986;136:241–242.
3. Rich MA, Keating MA, Snyder HM III, et al. Low transurethral incision of single system intravesical ureteroceles in children. J Urol 1990;144:120–121.
4. Hendren WH, Mitchell ME. Surgical correction of ureteroceles. J Urol 1979;121:590–597.
5. Scherz HC, Kaplan GW, Packer MG, et al. Ectopic ureteroceles: surgical management with preservation of continence—review of 60 cases. J Urol 1989;142:538–543.
6. Reitelman C, Perlmutter AD. Management of obstructing ectopic ureteroceles. Urol Clin North Am 1990;17:317–328.
7. Koyanagi T. Experience of ureterocele in children less than one year old. Urol Correspondence Club 1992;February 10:27–28.
8. Gotoh T, Asano Y, Nonomura K, et al. Cecoureterocele: experience of three cases with special reference to the relevance of endoscopic incision. J Pediatr Surg 1993;28:223–227.
9. Takeuchi I, Nonomura K, Kakizaki H, et al. Transurethral incision of ectopic ureterocele in infants. Jpn J Pediatr 1993;29:962–971.
10. Decter RM, Roth DR, Gonzalez ET. Individualized treatment of ureteroceles. J Urol 1989;142:535–537.
11. Monfort BG, Guys JM, Coquet M, et al.. Surgical management of duplex ureteroceles. J Pediatr Surg 1992;27:634–638.
12. Blyth B, Passerini-Gazel G, Camuffo C, et al. Endoscopic incision of ureteroceles: intravesical versus ectopic. J Urol 1993; 149:556–560.
13. Glassberg KI, Braren V, Duckett JW, et al. Report of the Committee on Terminology, Nomenclature and Classification, Section on Urology, American Academy of Pediatrics: suggested terminology for duplex systems, ectopic ureters and ureteroceles. J Urol 1984;132:1153–1154.
14. Nonomura K, Imanaka K, Nantami M, et al. Radioisotope split renal function and diuretic renogram in the surgical management of pelvo-ureteral junction. Jpn J Urol 1984;75:654–664.
15. Itoh K, Arakawa M. Re-estimation of renal function with $^{99m}$Tc-DTPA by the Gates' method. Jpn Nucl Med 1987;24:389–396.
16. Gotoh T, Koyanagi T, Matsuno T. Experience with transurethral incision of ureteroceles: endoscopic intervention revisited.Jpn J Urol 1988;79:1535–1543.
17. Eklöf O, Lohr G, Ringerts H, et al. Ectopic ureteroceles in the male infant. Acta Radiol 1970;19:145–153.
18. Yamashita T, Nonomura K, Kakizaki H, et al. Clinical characteristics of vesicoureteral reflux in children less than one year old. SIU 23rd World Congress Abs 1994;126:A198.
19. Rickwood AMK, Reiner I, Jones M, et al. Current management of duplex-system ureteroceles: experience with 41 patients. Br J Urol 1992;70:196–200.
20. Mor Y, Ramon J, Raviv G, et al. A 20-year experience with treatment of ectopic ureteroceles. J Urol 1992;147:1592–1594.

